# Bioavailability of Sulforaphane Following Ingestion of Glucoraphanin-Rich Broccoli Sprout and Seed Extracts with Active Myrosinase: A Pilot Study of the Effects of Proton Pump Inhibitor Administration

**DOI:** 10.3390/nu11071489

**Published:** 2019-06-29

**Authors:** Jed W. Fahey, Kristina L. Wade, Katherine K. Stephenson, Anita A. Panjwani, Hua Liu, Grace Cornblatt, Brian S. Cornblatt, Stacy L. Ownby, Edward Fuchs, Walter David Holtzclaw, Lawrence J. Cheskin

**Affiliations:** 1 Cullman Chemoprotection Center, Johns Hopkins University, Baltimore, MD 21205, USA; 2 Division of Clinical Pharmacology, Department of Medicine, Johns Hopkins University School of Medicine, Baltimore, MD 21205, USA; 3 Department of Pharmacology and Molecular Sciences, Johns Hopkins University School of Medicine, Baltimore, MD 21205, USA; 4 Center for Human Nutrition, Department of International Health, Johns Hopkins University Bloomberg School of Public Health, Baltimore, MD 21205, USA; 5 Nutramax Laboratories Consumer Care, Inc., Edgewood, MD 21040, USA; 6 Department of Health Behavior and Society, Johns Hopkins University Bloomberg School of Public Health, Baltimore, MD 21205 USA

**Keywords:** chemoprevention, crucifer, nutritional supplement, pharmacokinetics, pharmacodynamics

## Abstract

We examined whether gastric acidity would affect the activity of myrosinase, co-delivered with glucoraphanin (GR), to convert GR to sulforaphane (SF). A broccoli seed and sprout extract (BSE) rich in GR and active myrosinase was delivered before and after participants began taking the anti-acid omeprazole, a potent proton pump inhibitor. Gastric acidity appears to attenuate GR bioavailability, as evidenced by more SF and its metabolites being excreted after participants started taking omeprazole. Enteric coating enhanced conversion of GR to SF, perhaps by sparing myrosinase from the acidity of the stomach. There were negligible effects of age, sex, ethnicity, BMI, vegetable consumption, and bowel movement frequency and quality. Greater body mass correlated with reduced conversion efficiency. Changes in the expression of 20 genes in peripheral blood mononuclear cells were evaluated as possible pharmacodynamic indicators. When grouped by their primary functions based on *a priori* knowledge, expression of genes associated with inflammation decreased non-significantly, and those genes associated with cytoprotection, detoxification and antioxidant functions increased significantly with bioavailability. Using principal components analysis, component loadings of the changes in gene expression confirmed these groupings in a sensitivity analysis.

## 1. Introduction

Consumption of plant-based diets is widely recognized as an important component in reducing the risk of a variety of chronic diseases and in promoting optimal health across the lifespan [1,2]. Cruciferous vegetables, including broccoli, cabbage, cauliflower, Brussels sprouts, daikon, watercress, mustard, wasabi, and certain tropical vegetable species like *Moringa oleifera*, are especially protective due to their high contents of glucosinolates as well as flavonoids, carotenoids, and anthocyanins [3,4]. Glucosinolates are not protective in native form, but are converted to biologically active isothiocyanates (ITC) by the enzyme, myrosinase [4,5]. Myrosinase is present in cruciferous plant cells and is normally segregated from the glucosinolates until the cells are ruptured by chewing, freeze–thaw, plant pathogens, or other damage. This enzyme is also produced by some of the bacteria in our gut—our gastrointestinal microbiome [6,7,8,9]. When glucosinolates are ingested, myrosinase hydrolyzes them to ITC. These ITC are then metabolized rapidly by conjugation with glutathione followed by stepwise hydrolysis of those conjugates, leading ultimately to N-acetylcysteine derivatives (mercapturic acids). Collectively, these conjugates are known as dithiocarbamates (DTC) and can be quantified by the cyclocondensation reaction developed in our laboratory in the 1990s, and more recently refined [10].

Bioavailability of the protective isothiocyanates from cruciferous vegetables varies widely based upon mode of delivery, inter-individual and even intra-individual differences in biochemistry and in gut microbiome composition and performance, and a number of other factors. There is great confusion amongst the general public when these differences are used to support claims made about many of the plethora of dietary supplements that have proliferated since the potency of broccoli sprouts was discovered at Johns Hopkins University [2,11,12].

Sulforaphane (SF), an ITC from broccoli, is a highly promising agent currently under preclinical and clinical evaluation for disease prevention [13]. Oral SF from extracts of broccoli sprouts or seeds is converted to urinary DTC metabolites, and between 70% and 90% of the dose is consistently excreted in the urine of all subjects who have been studied [14]. SF is not very stable over time, especially in aqueous solution. SF-rich powders are made by drying broccoli sprout- or seed extracts (termed “BSE”). These BSE are extremely hygroscopic and the preparation of capsules (and appropriate controls) for use in clinical trials is challenging and very expensive. SF-rich BSE must be stored in a freezer to maintain potency.

On the other hand, administration of BSE rich in glucoraphanin (GR, the main glucosinolate in broccoli and broccoli sprouts), results in highly variable conversion of GR to SF metabolites. This conversion, which we also refer to as bioavailability, ranges from 1 to 40%, with a mean of about 10%. This huge range depends primarily on the specifics of the microbiome of the person ingesting it, as well as a host of other unknown anthropomorphic factors (e.g., genetics, diet, and metabolic differences between people [2,15,16,17]). GR-rich BSE, although hygroscopic, is stable when kept dry, and thus has a long shelf-life even at room temperature. Some GR may be excreted, either intact, or as non-ITC metabolites [18], and the majority of glucosinolates delivered to humans are not converted to ITC [2,5,11].

The idea that adding myrosinase to GR-rich BSE might enhance and stabilize SF production, led us to evaluate its administration to volunteers as freeze-dried broccoli sprouts which were added to fruit juice and allowed to incubate (autolyze) prior to consumption, and as fresh, ground broccoli seeds delivered in gel caps [11]. These GR-rich preparations were very stable at room temperature, and when a dose was ingested, endogenous myrosinase present in the seed powder efficiently hydrolyzed GR to SF and its DTC. Urinary DTC output ranged from 14 to 56% of dose, with mean bioavailability of about 35–40% [11]. These preparations were utilized in a number of clinical studies in the USA, and China, which continue to generate requests for the material [to be consumed] from the general public. Clearly, it was impractical to continue making these preparations at a biomedical research university, and it would have been unethical to supply them to the many consumers and former clinical study volunteers who requested them.

A number of these BSE dietary supplements rich in GR, SF, or GR + myrosinase (an “SF production system”) now appear to be continuing/enduring parts of the supplement marketplace. Many of them are capsules (gelatin or vegetarian capsules filled with powdered ingredients), with potentially very different pharmacokinetics than those reported [11]. In order to clear up some of the confusion about bioavailability, it is necessary to query representatives of each class of supplement mentioned above in order to ascertain whether the pharmacokinetics of SF delivery is as expected in the individuals consuming these tablets, pills, drinks, elixirs, and potions, once they are amended and formulated with the variety of common “inert” ingredients that the industry routinely employs [19]. Furthermore, although literature is starting to emerge highlighting the effects of SF delivery in subjects with a variety of syndromes and conditions (e.g., air pollution injury, aflatoxin exposure, COPD, asthma, diabetes, autism, schizophrenia, *Helicobacter* infection) [20,21,22,23,24], very little pharmacodynamics data have been published.

We approached a small number of dietary supplement companies selling BSE-based products that we had tested and found to contain consistent levels of GR or SF, to seek a supply of supplement for ongoing clinical investigations. We then conducted a pilot clinical study with one of those commercial BSE supplements which contains active myrosinase, in order to verify the rough equivalence of its yield (µmoles of SF delivered) with similar preparations produced at JHU, and we report on that study herein. Furthermore, we examined trends in bioavailability (reported as percent of dose excreted in the urine in 24 h, as SF and its metabolites) as stratified by sex, body weight, BMI, race, age, vegetable intake frequency, and bowel movement frequency. After confirming equivalence between the preparation (delivered in gel-caps) from JHU and the commercial supplement tablets, we examined the effects of gastric acidity on the enzymatic activity, under the assumption that normal and achlorhydric stomachs might have different effects on myrosinase activity during the time in which the bolus delivered by a supplement tablet transited the gastrointestinal system. This was evaluated by comparing delivery of enteric-coated and uncoated tablets, and by the administration of omeprazole, a proton pump inhibitor (PPI), with all subjects serving as their own control in a sequential dosing series of experiments. In addition to monitoring urinary excretion of SF and its DTC metabolites in full 24-h collections, we evaluated a variety of biomarkers thought to be associated with the mode of action of SF, in peripheral blood mononuclear cells (PBMCs) isolated from blood taken from subjects before and after an intervention with an oral GR + myrosinase supplement.

## 2. Materials and Methods

### 2.1. Design

Recruitment targeted healthy adults. Twenty participants were recruited for a brief, single dose pilot conversion study using the BSE plus myrosinase supplement, Avmacol^®^ (Nutramax Laboratories Consumer Care, Inc., Edgewood, MD, USA) (“Pilot Phase”; for demographics, see Table S1). After validating the fact that the commercial BSE supplement had roughly equivalent SF bioavailability to the laboratory-created food supplements we had for years been creating for clinical studies (only), we progressed to the “PPI Phase.” This phase was a more detailed examination of both enteric-coated, and uncoated commercial supplement tablet consumption by healthy volunteers before- and during a course of PPI therapy designed to ablate gastric acid production (for demographics see Table S2). All work was performed exclusively at Johns Hopkins University under the auspices of its IRBs (IRB0007742 and IRB00099644), and under IND 127,220 waiver from the US FDA.

**Figure 1:**
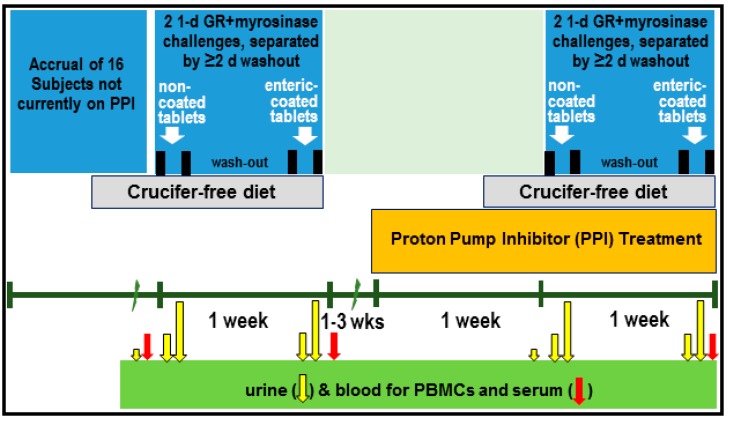
Design of PPI Phase clinical intervention.

For the PPI Phase, we recruited 16 subjects who had complaints about heartburn or gastroesophageal reflux disease (GERD) but were not taking PPIs. The study physician examined them, and if admitted into the study, they were provided omeprazole to be started only after an initial period of urine collection and ingestion of a single dose of non-coated supplement tablets followed 3 d later by ingestion of a dose of coated supplement tablets. For both phases, participants were instructed to initiate a cruciferous-free diet three days prior to the Avmacol^®^ dose and were given a list of foods to avoid. Instructions were also provided for completion of a contemporaneous diet diary (Pilot Phase, only) covering all food taken after dosing, to be maintained concurrent with urine collection. The tablets for participants in both phases were provided to be swallowed with bottled water at the beginning of the day (prior to the first meal of the day). Written instructions were given to each participant, and these were reinforced in person at the first study visit, for the [complete] urine collections: (1) an 8-h daytime collection (0–8 h), and (2) an evening/overnight 16-h collection (8–24 h). Dose administration, blood and urine sampling, and PPI (omeprazole) treatment are outlined in Figure 1.

### 2.2. Dose Preparation and Delivery

The BSE dietary supplement Avmacol^®^, containing GR and the active enzyme, myrosinase, was used for all phases of this investigation. Manufacturer certified GR content was ≥13 mg/tablet (≥30 µmol/tablet). Each lot was analyzed at JHU following tablet homogenization in a solvent mix comprised of equal parts of dimethyl sulfoxide, acetonitrile, dimethyl formamide and water according to published methods [10,25,26,27]. Measured GR content, later used to assess conversion efficiency, was 35.2, 46.2, and 46.0 µmol/tablet for production lots RD1015-10 (Pilot Phase, uncoated), RD0416-01 (PPI Phase, uncoated), and RD0416-03 (PPI Phase, enteric-coated), respectively. Dose was 6 tablets per day per subject for the Pilot Phase, estimated to deliver about 211 µmol/subject/day of GR, and 8 tablets per day per subject for the PPI Phase, estimated to deliver about 369 µmol/subject/day of GR.

### 2.3. Urine Collections

A baseline urine collection was obtained from each subject at the end of the 3-day crucifer-free diet period, immediately prior to ingestion of the first BSE dose. After ingesting the dose in the presence of a study team member, participants were required to collect their urine for the following 24 h, divided into two time periods: an 8 h collection from 08:00 to 16:00 and a 16 h collection from 16:00 to 08:00 the next day. Labeled bottles were provided to each subject for this purpose. Participants were not required to refrigerate urine as collected, but were asked not to leave them in extreme environments (i.e., in a hot car in the summertime). We have previously confirmed this to sufficiently protect sample integrity, and no additional stabilizers need be added to samples [10,11,12,15,19,23,28]. The pre- and post-dose urine volumes were measured, and aliquots in triplicate were frozen at −80 °C until analyzed.

### 2.4. Assay of Urine ITC/DTC

The ITC content of the urine samples was determined by the cyclocondensation reaction-HPLC assay [10]. This assay effectively captures SF and all its metabolites as DTC with a 24-h complete urine collection, as previously described [10,25,27]. Briefly, the method is based upon the knowledge that ITC are metabolized in mammals principally by the mercapturic acid pathway. An initial conjugation with glutathione (GSH) promoted by glutathione transferases gives rise to the corresponding GSH–ITC conjugates. These undergo further enzymatic modifications to give rise sequentially to the cysteinylglycine–, cysteine– and N-acetylcysteine–ITC conjugates, all of which are DTC. Nearly all ITC and DTC react quantitatively with the vicinal sulfhydryl groups of 1,2-benzenedithiol to give rise to a cyclic condensation product (1,3-benzodithiole-2-thione), which has properties that are highly suitable for spectroscopic quantification (A_m_ = 23,000 M^−1^ cm ^−1^ at 365 nm). The cyclocondensation product is stable, and can be separated and quantified by HPLC. Because all known ITC conjugates from human tissues are DTC which react with 1,2-benzenedithiol, the cyclocondensation assay is ideal for determining the total DTC metabolites [10]. Full recovery of ITC metabolites on a molar basis has been demonstrated by Ye et al. [10] and others.

Duplicate 200 μL aliquots from the pre-dose urines and 20–200 μL aliquots from the post-dose urines were used. Peak areas of the cyclocondensation product (CCP) of the urine samples were quantitated by comparison to injections of known amounts of a CCP standard. Pre-dose urine DTC values are measured as nmol/mL, and post-dose DTC values are measured as μmol per 8 h and μmol per 16 h. A sum of the two values is created (μmol per 24 h), and conversion efficiency is reported as {the sum of SF and its DTC metabolites excreted in 24 h (in µmol) as measured by the cyclocondensation reaction divided by µmol per dose of GR}.

### 2.5. Blood and PBMC Collection (PPI Phase, Only)

Three blood samples were collected: at baseline, one on the morning after dosing of the enteric-coated supplement, and on the morning after dosing of the enteric-coated supplement with omeprazole (see Figure 1). Blood was collected in 8 mL Vacutainer^®^ CPT^™^ tubes (Becton, Dickinson and Company, Franklin Lakes, NJ, USA) and processed for PBMC isolation according to the instructions from the manufacturer. PBMC pellets were frozen at −80 °C until analyzed.

### 2.6. Total RNA Isolation, Reverse Transcription-PCR, and Quantitative PCR

Total cellular RNA was isolated from PBMCs using the RNeasy mini kit (Qiagen, Valencia, CA, USA). RNA quantity and quality were measured with the NanoDrop 2000 spectrophotometer (Thermo Fisher Scientific, St. Louis, MO, USA) and complementary DNAs (1 µg) were synthesized using the SuperScript III First Strand Synthesis Kit (Invitrogen). Real-time PCR was performed using the CFX Connect Real-Time PCR Detection System (Bio-Rad Laboratories, Hercules, CA, USA) with RT^2^ qPCR Primer Assays (Qiagen). Relative expression values of molecular markers were normalized to ribosomal protein *RPLPO*. Amplification specificity was determined by performing melt curve analysis to detect non-specific amplification artifacts.

### 2.7. Analyses

Analysis of data obtained from urine collections was prescreened for completeness. Values were censored from further analyses due to either: (a) unexpectedly high baseline (*Subject 20, Pilot Phase*), (b) apparent incomplete urine collection (i.e., suspiciously low urine volumes), (c) subject illness (*Subject 12, PPI Phase*), or (d) total 24-h excretions below 5% (*Subjects 4, 5, 8, PPI Phase*). All censored values not used in subsequent plots or calculations in Table 1 and Table S1, are flagged in red. All conversion values reported are presented on a molar equivalence basis (moles of GR, MW-436, to moles of SF, MW-177) adjusted based on actual µmoles of GR in the tablets given to each subject (see Section 2.2).

Bioavailability (conversion) data were compared between treatments using paired *t*-tests. A random intercept, linear mixed effect model was used to evaluate within-cluster and between-cluster covariate effects to determine whether there were any significant correlations between DTC excretion magnitude and BMI, stool frequency, age, race, or gender. Oneway ANOVA was used to assess significance of BMI and body weight on conversion.

Blood samples were (only) drawn at the onset of the study period, then following the two “pre-ppi”, and again following the two “on-ppi” doses. The biomarker values assessed in the PBMCs from the latter two blood draws were averaged and compared to those in the initial (pre-intervention) blood draw. This is reported in the legends to relevant figures as “means following single pre- and post-omeprazole delivery of an oral BSE dose containing GR and active myrosinase” for sake of brevity.

Principal component analysis (PCA) was performed on the gene biomarkers and set to three components based on eigenvalues and visual inspection of the scree plot. All statistical manipulations were performed on either Excel (Microsoft, Redmond, WA) or Stata versions 11.2 and 14.0 (StataCorp, College Station, TX, USA).

## 3. Results

**Figure 2:**
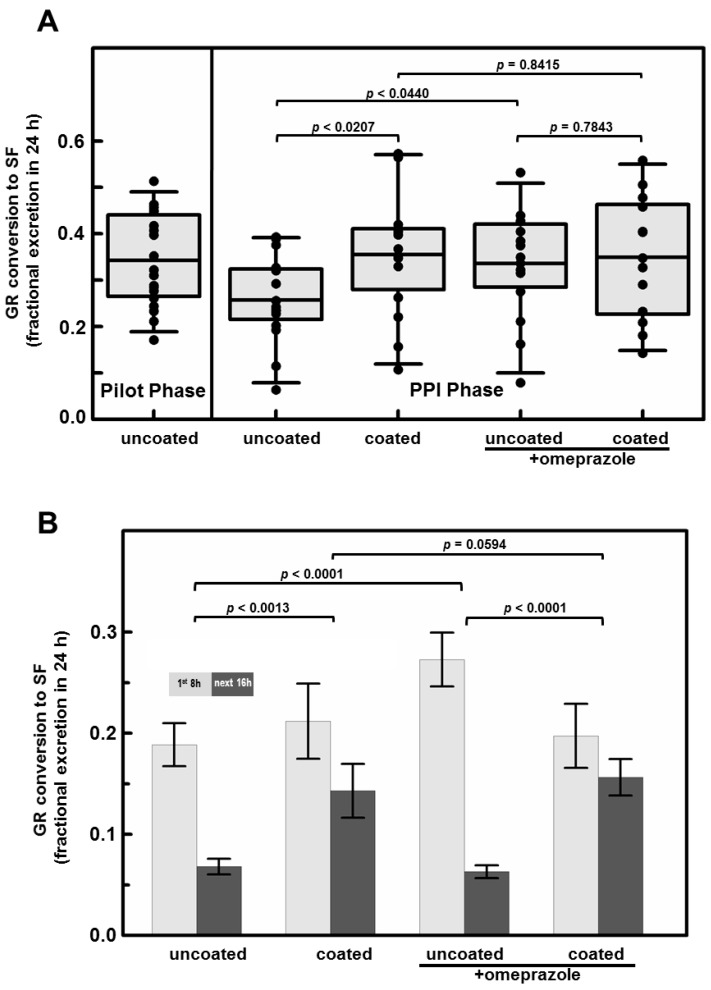
(**A**) Bioavailability following a single oral dose of a broccoli seed and sprout extract (BSE) containing GR and active myrosinase, as a function of treatment. There was a significant difference by paired *t*-test between uncoated and coated administration, prior to omeprazole administration (*p* < 0.0207), and a significant increase in bioavailability from uncoated tablets given after- compared to before- PPI administration (*p* < 0.0440). Error bars represent the 5th and 95th percentile of the confidence interval around the mean, and boxes bracket the middle 50% of the distribution range. (**B**) Excretion of SF metabolites following a single oral dose of BSE containing GR and active myrosinase, in urine collected over 1st 8 h post-dose (0–8 h) and the following 16 h (8–24 h). Paired *t*-tests for differences between 1st 8 h and next 16 h were highly significant for treatments in which tablets were uncoated (*p* < 0.0001), but not significant for treatments in which tablets were enteric-coated (*p* = 0.0594). Tablet coating significantly delayed conversion both prior to omeprazole treatment (*p* < 0.0013) and following omeprazole treatment (*p* < 0.0001). Error bars represent S.E.M.

**Figure 3:**
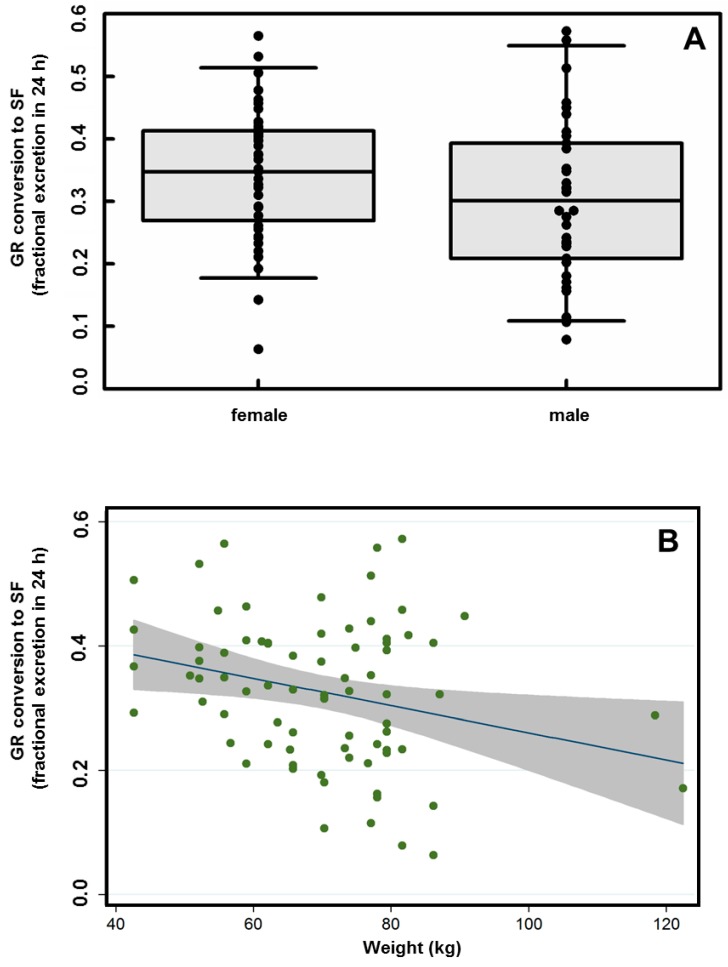
Excretion of SF metabolites following a single oral BSE dose containing GR and active myrosinase. (**A**) Relationship between sex of participant and bioavailability (NSD). (**B**) Relationship between body weight and bioavailability. Shaded region represents 95% C.I. around fitted line (F_1,74_ = 5.83, *p* < 0.018).

Conversion of GR to SF was reported as percent of dose excreted, and was normalized based on a molar equivalence basis (moles of GR to moles of SF plus its metabolites as determined in the cyclocondensation reaction). We have performed extensive analyses of tablets from each lot used, to confirm precise GR content, myrosinase activity, and complete absence of any pre-formed SF. Although there were differences in GR content per tablet across the three manufacturing lots utilized in the study, there were no significant differences in percent conversion (GR to SF, normalized on a molar basis), between lots. Conversion was virtually the same in both phases of this study with no significant difference between male and female subjects (Figure 2 and Figure 3). Total excretion was 34.3% of dose (standard deviation, 9.7% of dose) in the Pilot Phase and 32.8% of dose (standard deviation, 4.7% of dose) in the PPI Phase (Table 1 and Table S1). Most conversion occurred within the first 8 h post-dose in all treatments and conditions (Figure S1) and in almost every one of the 83 individual-by-treatment combinations (Table 1 and Table S1), with an overall mean conversion efficiency of 32.7% for all treatments combined.

The primary hypothesis being examined in this study was whether the acidity of the stomach would have an effect upon the activity of myrosinase, co-delivered with GR. There was a 28% reduction in conversion of GR to SF (*p* < 0.0207) comparing uncoated to coated tablets delivered to the same subjects prior to taking omeprazole (Figure 2A). Delivering GR with active myrosinase in an enteric-coated tablet yielded a mean conversion of 36% regardless of omeprazole use (subjects taking omeprazole were presumed to have an achlorhydric stomach). Subjects consuming uncoated tablets (presumed to dissolve rapidly in the stomach), and also taking omeprazole, had similar conversion (33.6%). However, when the same subjects consumed uncoated tablets before taking omeprazole, so their stomachs were still highly acidic, their mean conversion was (25.7%) (Figure 2A). Interestingly, delivering the mix of active myrosinase enzyme plus GR in coated tablets also delayed conversion so that twice the conversion occurred in the 8- to 24-h time period (15.5%) as did with the uncoated delivery system (6.6%) (Figure 2B; Table 1). By paired *t*-tests, the differences between the overall mean of the first 8 h (21.7%) and the overall mean of the next 16 h (10.7%) excretion were highly significant regardless of omeprazole status. These differences between collection time were significant when participants were either not taking omeprazole (*p* < 0.0013) or taking omeprazole (*p* < 0.0001) regardless of tablet coating. Differences in conversion were also significant for uncoated tablets regardless of PPI status (*p* < 0.0001), but not for coated tablets (*p* = 0.0594), suggesting that release of tablet contents occurred later in transit through the gastrointestinal system. Enteric coating appeared to enhance SF delivery in acid-producing stomachs, but when subjects consumed coated tablets, they reported far more adverse events than when those same subjects consumed uncoated tablets (Table 2).

There were no significant effects of sex (30.1 and 34.9% of dose respectively) on conversion efficiency (Figure 3A). There were also no significant effects of ethnicity, age, BMI, vegetable consumption, or bowel movement frequency or quality (Figures S2–S6). There was, however, a significant reduction in conversion (from 38.5% to 21.1%) with increasing participant body weight ranging from 42.6 to 122.5 kg (F_1,74_ = 5.83, *p* < 0.018; Figure 3B).

As with any clinical study, subject compliance with the protocol is always an issue. In this study, there were two areas of concern vis-à-vis compliance and its measurement: The first is adherence to the dietary restrictions. This was evaluated by examining the pre-dose DTC value; a high pre-dose value suggests a failure to adhere to the dietary restrictions, likely leading to 24 h DTC values that may be elevated and unreliable. Diet diaries were used to assist in the diagnosis of subject compliance only in the Pilot Phase of the study. No overt digressions were noted in an inspection of the diaries. Some participants did note that they “picked out” the banned foods when presented as a mixed dish in an effort to adhere to the study diet. The second major area of concern regarding compliance is ensuring that there has been complete collection of the 24 h urine. A complete 24 h urine collection has been demonstrated in multiple studies to be adequate to capture essentially all of the DTC produced from ingestion of either GR or SF [10,11,14,15,16,17,23,28]. Conversion efficiency values of a limited number of urine collections were censored as indicated in Section 2. Furthermore, the use of concomitant drugs (prescribed, OTC, or illicit), cannot be ruled out as a possible confounding factor. The study design was such that some medications were permitted, but we insisted that they be recorded, and vetted by the study physician and study team. Those reported drugs were: *Subject 1* took 1 naproxen for a headache at ~5–6 PM during Dose 3 and 1 naproxen at 7 PM during Dose 4; *Subject 2* suffers from migraines and takes nortriptyline once or twice a month. Nortriptyline and BC^®^ powder were taken during Dose 1 by this participant; *Subject 6* used ginger beer to calm her stomach following Dose 1 and baking soda after Doses 2 and 4 subsequent to vomiting breakfast ~11 am; *Subject 7* took Lipitor^®^ on a regular basis; *Subject 10* took birth control pills and Claritin^®^ on a regular basis; *Subject 12* took 2 doses each of Benadryl^®^ and Dayquil^™^ for fever/cold during Dose 3; *Subject 14* took Vyvanse^®^ regularly and 2 tablets regular strength TylenolPM^®^ at 3 PM the day before and 2 tablets of Advil Migraine^®^ at 6 PM during Dose 3; *Subject 15* used steroid ointment on eczema when it flared, and started birth control pills before 3rd dose.

**Figure 4:**
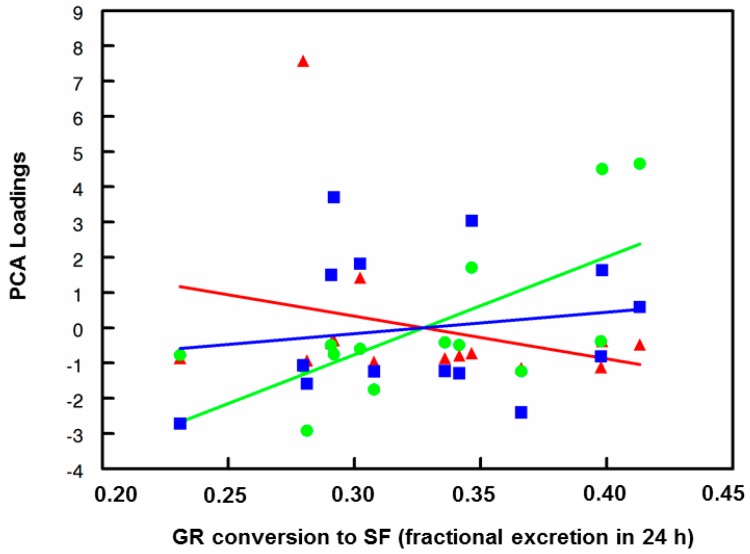
Principal component analysis (PCA) loadings and sulforaphane bioavailability (means following single pre- and post-omeprazole delivery of an oral BSE dose containing GR and active myrosinase). PC1(▲), PC2 (●), PC3(■).

Twenty genes that are potential pharmacodynamics biomarkers for SF were evaluated by real-time PCR. They are: NQO1 (NAD(P)H:quinone oxidoreductase-1), GCLC (glutamate-cysteine ligase catalytic subunit), GCLM (glutamate-cysteine ligase modifier subunit), HSP27 (heat shock protein 27), HSP70 (heat shock protein 70), HO-1 (HMOX-1; heme oxygenase-1), HDAC3 (histone deacetylase-3), IL-1β (interleukin-1β), SOD2 (superoxide dismutase-2), IL-2 (interleukin-2), IL-10 (interleukin-10), IL-6 (interleukin-6), IL-8 (interleukin-8), COX-2 (cyclooxygenase-2), SMPD1 (acidic sphingomyelinase), SLC7A11 (xCT; cysteine/glutamate antiporter), IFNγ (interferon-γ), CAT (catalase), AKR1c1 (aldo-keto reductase family 1 member c1), and AKR1B10 (aldo-keto reductase family 1 member b10). These markers were chosen based upon the potential of SF to upregulate the Nrf2 and heat shock response (HSR) pathways, and to inhibit the NF-ÍB related inflammatory pathway, among other endpoints, which have been reviewed previously [13,20,21,23,29]. Real-time PCR was used to compare fold-changes of gene expression in PBMCs post-intervention with pre-intervention as baseline (Figure S7). Baseline expression levels were set at 1.0, and changes in expression were analyzed for principal components and set to three components based on a scree plot of eigenvalues (data not shown). Changes in four of the 20 genes evaluated were not explained by the three components, but these three components explained 70% of the variance, with 58% explained by the first two components (Figure S8 and Table S3). Components and loadings are presented in Table S3, and PCA plots are shown in Figure 4 and Figure S9. Correlations between components (groups of candidate biomarker genes) and bioavailability (conversion of GR to SF) as well as subject anthropometric characteristics are presented in Figure 4 and Figures S10–S13. Biomarkers of inflammation and immune response (clustering in PCA analysis as group PCA1) declined with increasing bioavailability (NSD), whereas biomarkers of the cytoprotective, detoxification, and antioxidant responses (clustering in PCA analysis as group PCA2) increased their expression significantly with increasing bioavailability of SF (F_1,12_ = 10.3; *p* < 0.0075; see Figure 4). When inspected at the level of the individual gene expression (20 such biomarkers were evaluated), the trends were also apparent, although the subject number was not adequate to make robust conclusions about these trends on a gene-by-gene basis, and Subject 1 was clearly an outlier from the perspective of amplitude of change. This subject reported taking naproxen for headaches prior to each dose, but did not report other concurrent illness. This subject had extraordinarily high IL-1β, IL-8, and COX2, gene expression values at both time periods that went into the average values used to create eigenvalues. These 3 genes were all in Component 1 in the PCA analysis.

## 4. Discussion

We use excretion of total ITC and their metabolites (DTC) (expressed as a percent of GR dose), as a proxy for the bioavailability of GR delivered in broccoli extract. This is consistent with the way we and others have reported bioavailability in the past [6,11,14,15,28,30], and consistent with the fact that the extracts being given to volunteers are almost exclusively composed of GR, which is converted to SF (in this case, with a catalytic boost from myrosinase co-delivered in the dietary supplement being used). We know that nitriles and other derivatives can be produced from glucosinolates under non-physiologic conditions, perhaps even including the digestive aberrations which lead to GERD and the need for proton pump inhibitors [3,4,5]. However, the present study was designed specifically to evaluate a potential reduction of myrosinase activity due to the acidity in the stomach, and as such, we only delivered single, acute doses for each of the sets of conditions being examined, and did not perform exhaustive analysis of all possible metabolites of GR. When we evaluated biomarkers, particularly the inflammatory biomarkers, there were heterogeneous responses. Many individuals demonstrated post-dose reductions in these markers, but some had significant increases. Since elimination of cruciferous vegetable consumption for three days immediately prior to dosing was required by our protocol, perhaps in some individuals the gastrointestinal microbiome dealt with the reintroduction of GR and SF (at the relatively high dose which we delivered), as a potentially toxic challenge, and reacted by upregulating pro-inflammatory pathways. Regular crucifer consumers could well have had altered microbiomes, and likewise, different microbiomes appear to react differently to a phytochemical challenge [31]. Thus, a follow-up study that is designed to evaluate the potential “hormetic” or “hormesis” effects of SF [32] would require a longer-term exposure and sampling of biomarkers. Such work has been done by us and others with children with autism spectrum disorder and other conditions [33,34,35], but a more critical evaluation of biomarkers would be in order.

In a previous, similarly designed study using material made by us for the study, and without added myrosinase, gender was marginally significant (males converted more than females), and there was no discernable effect of any of the other demographic parameters examined, on bioavailability of GR [6]. In the present study, none of the demographic parameters examined proved to be significant, with the exception of body weight. Interestingly, we did see a difference in the conversion of GR to SF delivered in uncoated tablets, between the Pilot and PPI Phases. These were different populations of volunteers—though both were “healthy”, those who volunteered for the PPI Phase were seeking relief from heartburn or GERD-like symptoms. It is entirely possible that their gastric acidity was greater (lower pH or greater acid production), thus leading to greater destruction of myrosinase, which is what is suggested by our data (see Figure 2A).

Among the hypotheses that have long been implicit in the work we have done on bioavailability have been the presumptions that: (a) consumption of glucosinolate-rich food or supplements might have an effect on the populations of myrosinase-containing bacteria in the gastrointestinal tract and/or their production of myrosinase; and (b) there may be a selective antibiotic effect of regular delivery of SF to the gastrointestinal tract. If either one of these hypotheses had any validity: (a) one might expect changes in the capacity for conversion (e.g., in bioavailability of a bolus of GR); (b) one might select for higher bioavailability by continued ingestion of GR or other glucosinolate-containing cruciferous vegetables; (c) this might present an opportunity for the development of “probiotic” supplements designed to enhance gut myrosinase activity; (d) it might similarly present an opportunity for the development of “prebiotic” supplements meant to selectively encourage myrosinase-producing species of gut-colonizing bacteria. To date, none of these hypotheses seem to have had promise: We previously confirmed that when the gut bacteria were removed following antibiotic treatment and mechanical cleansing, myrosinase activity was lost, and re-appeared concomitant with repopulation of the lower bowel [30]. Rat studies, some using germ-free colonies, have examined the bacterial species most likely responsible [8]. In vitro studies of human gut isolated bacterial strains that when incubated with glucosinolates confirmed myrosinase-like activity [7]. In these experiments, ex vivo human gut bacteria isolated from fecal samples of glucosinolate high and low converters, demonstrated no difference in bacteria population, but an initial difference in conversion capacity. Others attempted to influence the gut bacteria by adding the prebiotic inulin, and whereas they increased *Bifidobacterium* populations, they failed to increase glucosinolate conversion capacity [18]. A month-long intervention with 200 µmol/day of GR supplied as fresh broccoli sprouts containing active myrosinase resulted in reduced constipation and reduced levels of *Bifidobacterium* in stool compared to control subjects [36].

Upon close inspection of medications being taken by subjects, we did not see any indication of correlations between medication use and conversion efficiency. Nonetheless, an itemization of medications used by our subjects is included as part of the Results, and is annotated on Figure S7. Similarly, lack of correlation was observed for bowel and dietary habits, gender, BMI, age, and ethnicity, although the study was not powered to detect such differences. We did observe a significant negative correlation between body weight and conversion efficiency. If, due to some abnormality of digestion that we have never seen before in many bioavailability studies, there is extremely low dissolution of the enteric-coated tablets, then it is conceivable that we are missing the results of full conversion in some of these subjects, and that more SF metabolites might have been collected had we taken longer full urine collections. However, much previous published work [10,11,14,28] supports the need to collect only 24 h of urine in order to almost completely capture conversion.

There was a dark side to the apparently delayed myrosinase activity which appeared to follow delivery of coated tablets to human volunteers. Tablet coating resulted in far more adverse events (AE), which we conjecture was due to myrosinase activity occurring farther along in the gastrointestinal tracts (e.g., in the small intestine rather than stomach) or possibly an adverse reaction to one of the additional ingredients used for enteric coating. It should be noted that this was not a completely healthy population since they enrolled in the study based upon their self-reported GERD or heartburn. The larger number of AE complaints and their greater severity present a cautionary note to employing a tablet enteric-coating strategy for trying to avoid the potentially damaging effects of gastric acidity on the functioning of a delivered active myrosinase. Further, there is no evidence to our knowledge of myrosinase activity in the oral cavity, esophagus, or stomach, and it is quite clear that humans have no endogenous myrosinase or myrosinase-like activity. Thus, GR conversion is mediated in the small or large intestine by the resident microflora, or throughout the gastrointestinal tract only following administration of exogenous myrosinase, as is the case with the combination product being delivered in this intervention.

## 5. Conclusions

With these observations in mind, we conclude that bioavailability of SF from GR-rich BSE that also contain active myrosinase, is very similar whether they are given to healthy subjects with normal acid stomachs, or to subjects in whose stomachs acid production has been medically ablated. The intervention used in this study was orally delivered commercial supplements. These supplements were supplied both as uncoated (“non-acid-protected”) tablets, or identical tablets that had been enteric-coated with an acid-resistant outer layer to prevent dissolution or release of contents in the stomach. Our results suggest that although this treatment improved bioavailability by 28% when subjects had normal gastric acidity, and had no effect when subjects took a proton pump inhibitor, there were a substantial number of adverse events reflecting abdominal discomfort and indigestion associated with consumption of the coated tablets. There were negligible effects of a variety of anthropometric factors (e.g., age, sex, ethnicity, BMI, vegetable consumption, bowel movement frequency and quality, or concurrent medications) on conversion efficiency, and a marginally inverse correlation between body mass and conversion efficiency. Expression of genes associated with inflammation decreased non-significantly, and those associated with cytoprotection, detoxification, and antioxidant functions increased significantly with bioavailability. Thus, further study is indicated if a commercial supplement manufacturer should decide to produce such coated tablets for widespread use. Likewise, the study of chronic (e.g., daily) ingestion of these supplements would be expected to further elucidate pharmacodynamics effects that could only be suggested, based on the dosing regimen used in this study.

## Figures and Tables

**Table 1:** PPI Phase conversion efficiency (SF bioavailability) normalized on a molar basis to the dose received. Numbers in *red italics* were censored from any further treatment of the data (and are not included in column- or row-averages in this table), due to apparent incomplete urine collection or extraordinarily low output as further described in Section 2.7.

	No PPI						PPI								
	Uncoated			Coated			Uncoated			Coated			Overall Means		
Subject #	8 h	16 h	24 h (Sum)	8 h	16 h	24 h (Sum)	8 h	16 h	24 h (Sum)	8 h	16 h	24 h (Sum)	8 h	16 h	24 h
1	18.71%	5.49%	24.20%	10.99%	4.66%	15.65%	10.99%	5.21%	16.20%	25.35%	30.43%	55.78%	16.51%	11.45%	27.96%
2	15.08%	5.14%	20.22%	0.44%	32.51%	32.95%	32.61%	5.81%	38.42%	0.27%	20.59%	20.86%	12.10%	16.01%	28.11%
3	28.88%	8.67%	37.55%	10.98%	28.79%	39.77%	45.89%	7.28%	53.17%	16.48%	18.27%	34.75%	25.56%	15.75%	41.31%
4	4.18%	2.17%	6.35%	*0.09%*	*0.18%*	*0.27%*	31.79%	8.68%	40.47%	9.74%	4.51%	14.25%	15.24%	5.12%	20.36%
5	0.74%	10.73%	11.47%	2.84%	32.41%	35.25%	35.84%	8.10%	43.94%	*0.08%*	*2.78%*	*2.86%*	13.14%	17.08%	30.22%
6	17.33%	11.88%	29.21%	31.56%	5.15%	36.71%	35.75%	6.87%	42.62%	39.55%	11.00%	50.55%	31.05%	8.73%	39.77%
7	15.75%	3.49%	19.24%	33.13%	8.83%	41.96%	31.54%	5.91%	37.45%	32.18%	15.59%	47.77%	28.15%	8.46%	36.61%
8	30.14%	2.54%	32.68%	16.49%	24.39%	40.88%	17.87%	3.22%	21.09%	*0.08%*	*1.11%*	*1.19%*	21.50%	10.05%	31.55%
9	31.71%	7.57%	39.28%	20.08%	6.16%	26.24%	23.28%	4.24%	27.52%	9.58%	13.65%	23.23%	21.16%	7.91%	29.07%
10	18.96%	5.23%	24.19%	34.79%	5.65%	40.44%	28.01%	5.61%	33.62%	27.49%	12.83%	40.32%	27.31%	7.33%	34.64%
11	16.35%	9.21%	25.56%	9.63%	12.41%	22.04%	34.77%	7.99%	42.76%	19.95%	12.75%	32.70%	20.18%	10.59%	30.77%
12	18.32%	5.24%	23.56%	23.53%	11.26%	34.79%	*3.70%*	*0.29%*	*3.99%*	*13.49%*	*4.84%*	*18.33%*	20.93%	8.25%	29.18%
13	25.63%	6.41%	32.04%	4.35%	6.34%	10.69%	26.51%	4.99%	31.50%	2.42%	15.65%	18.07%	14.73%	8.35%	23.08%
14	17.18%	5.62%	22.80%	35.86%	5.28%	41.14%	26.92%	5.29%	32.21%	26.71%	13.74%	40.45%	26.67%	7.48%	34.15%
15	26.52%	12.37%	38.89%	38.53%	17.93%	56.46%	22.31%	12.61%	34.92%		29.02%	29.02%	29.12%	17.98%	39.82%
16	16.07%	7.33%	23.40%	44.52%	12.71%	57.23%	5.14%	2.75%	7.89%	27.06%	18.73%	45.79%	23.20%	10.38%	33.58%
Mean -->	18.85%	6.82%	25.67%	21.18%	14.30%	35.48%	27.28%	6.30%	33.59%	19.73%	16.67%	36.41%			
Mean -->	30.57%						35.00%								
Mean -->	32.79%														

**Table 2:** Adverse events reported (PPI Phase).

Treatment	Dose	Constipation	Loose BM	Abdominal Pain	Bloating	Excess Gas
No PPI	uncoated	1	3	4	5	4
No PPI	coated	0	8	11	4	4
+ PPI	Uncoated ^a^	0	4	4	4	4
+ PPI	coated	1	9	10 ^b,c^	4	5

^a^ One subject had a cold/fever, reported concurrent with dose 3. ^b^ One subject vomited following dose 4. This was relieved with sodium bicarbonate. ^c^ One subject reported abdominal pain ~2 h post dose 4 so bad that he was “lying on the bathroom floor.” Pain was ranked a 7 on a 1–10 scale and lasted about 1.5 h.

## Supplementary Materials

The following are available online at https://www.mdpi.com/2072-6643/11/7/1489/s1, Figure S1. Twenty-four-hour SF metabolite excretion following a single oral dose of BSE containing active myrosinase. Figure S2. Relationship between self-identified ethnicity and bioavailability. Figure S3. Relationship between age and bioavailability. Figure S4. Relationship between body mass index (BMI) and bioavailability. Figure S5. Effect of prior self-reported vegetable consumption on 24 h excretion of sulforaphane metabolites following a single oral dose of BSE containing glucoraphanin and active myrosinase. Figure S6. Relationship between self-reported (A) bowel movement frequency and (B) bowel movement quality on 24 h excretion of sulforaphane metabolites following a single oral dose of BSE containing glucoraphanin and active myrosinase. Figure S7. Mean gene amplification: 20 genes for each of 16 subjects, grouped according to PCA results. Figure S8. PCA scores for components 1 and 2. Figure S9. Gene amplification (fold-change from baseline) and sulforaphane bioavailability. Figure S10. PCA loadings and sulforaphane bioavailability by age of subject. Figure S11. PCA loadings and sulforaphane bioavailability by self-identified ethnicity of subject. Figure S12. PCA loadings and sulforaphane bioavailability by sex of subject. Figure S13. PCA loadings and sulforaphane bioavailability by self-identified body mass of subject. Table S1. Pilot Phase demographics and results. Table S2. PPI Phase demographics. Table S3. Gene expression loadings by component following interventions, compared to those at baseline.

## Abbreviations

BMI	body mass index
BSE	broccoli sprout and seed extract
DTC	dithiocarbamates
GR	glucoraphanin
HSR	heat shock response
ITC	isothiocyanates
PBMC	peripheral blood mononuclear cells
PCR	polymerase chain reaction
PPI	proton pump inhibitor
SF	sulforaphane
