# Supplementary material for: Bioavailability of Sulforaphane Following Ingestion of Glucoraphanin-Rich Broccoli Sprout and Seed Extracts with Active Myrosinase: A Pilot Study of the Effects of Proton Pump Inhibitor Administration
Source: Nutrients. 2019 Jun 29;11(7):1489. doi: 10.3390/nu11071489 (PMC6682992; doi:10.3390/nu11071489)

SUPPORTING INFORMATION:

Bioavailability of sulforaphane following ingestion of glucoraphanin-rich broccoli sprout and seed extracts with active myrosinase: A pilot study of the effects of proton pump inhibitor administration

Jed W. Fahey[1,2,3,4,*], Kristina L. Wade[1,3], Katherine K. Stephenson[1,3], Anita A. Panjwani[1,4], Hua Liu[1,3], Grace Cornblatt[5], Brian S. Cornblatt[5], Stacy L. Ownby[5], Edward Fuchs[2], Walter David Holtzclaw[1,3], Lawrence J. Cheskin[6,‡]

[1] Cullman Chemoprotection Center, Johns Hopkins University, Baltimore, Maryland, USA
[2] Johns Hopkins University School of Medicine, Department of Medicine, Division of Clinical Pharmacology, Baltimore, Maryland, USA
[3] Johns Hopkins University School of Medicine, Department of Pharmacology and Molecular Sciences, Baltimore, Maryland, USA
[4] Johns Hopkins University Bloomberg School of Public Health, Department of International Health, Center for Human Nutrition, Baltimore, Maryland, USA
[5] Nutramax Laboratories Consumer Care, Inc., Edgewood, Maryland, USA
[6] Johns Hopkins University Bloomberg School of Public Health, Department of Health Behavior and Society, Baltimore, Maryland, USA
‡ Current Address*: George Mason University, Department of Nutrition and Food Studies*

*Address for correspondence: Dr. Jed W. Fahey, Director, Cullman Chemoprotection Center, Johns Hopkins University, 855 N. Wolfe St, Suite 625, Baltimore, Maryland, USA 21205; jfahey@jhmi.edu

Supplementary Results and Discussion.

An evaluation of any specific gene (e.g. NQO1, which encodes for a prototypical detoxification and cytoprotective enzyme) must be tempered by knowledge that only a single dose was given and gene effects were evaluated a full 24 h later in systemic blood (PBMCs). This would not have permitted us to carefully inspect or query the temporal induction or inhibition events that would be expected to occur within a much shorter time period following the dose.

A most intriguing study appeared while this investigation was underway [1]. These investigators examined a few of the same biomarkers and anthropometric factors that we report upon herein and although not directly comparable (e.g. measured in plasma rather than in PBMCs, and a chronic exposure study) they showed significant reduction in IL-6 and CRP in overweight subjects while consuming BSE with active myrosinase.

Supplementary References

1. López-Chillón, M.T.; Carazo-Díaz, C.; Prieto-Merino, D.; Zafrilla, P.; Moreno, D.A.; Villaño, D. Effect of long term consumption of broccoli sprouts on inflammatory markers in overweight subjects. *Clin. Nutr.* **2019**, 38, 745-752.

Table S1. Pilot Phase demographics and results. BSE supplement dose = 211.2 µmol glucoraphanin. Numbers in *red italics* have been censored from any further treatment of the data (and are not included in column-averages in this table), due to apparent incomplete urine collection.

					Excretion reported as µmol and (% of dose))		
Participant	Sex	Race	Age	BMI	8 hr	16 hr	Total (8+16)
01	F	Cauc	40	24.0	39.31 (25.6%)	19.19 (3.2%)	58.50 (28.8%)
02	F	Cauc	34	18.1	56.58 (26.8%)	17.80 (8.4%)	74.38 (35.2%)
03	F	AA	27	27.3	25.86 (12.2%)	18.67 (8.8%)	44.53 (21.1%)
04	F	AA	40	24.7	38.53 (18.2%)	10.73 (5.1%)	49.26 (23.3%)
05	M	Cauc	56	No Report	64.14 (30.4%)	30.91 (14.6%)	95.05 (45.0%)
06	M	AA	44	No Report	40.33 (19.1%)	19.77 (9.4%)	60.10 (28.5%)
07	M	Cauc	49	35.6	13.87 (6.6%)	22.15 (10.5%)	36.02 (17.1%)
08	F	Cauc	69	20.8	25.21 (11.9%)	26.29 (12.4%)	51.50 (24.4%)
09	F	AA	53	29.4	68.43 (32.4%)	19.55 (9.3%)	87.98 (41.7%)
10	M	AA	47	37.4	54.09 (25.6%)	6.83 (3.2%)	60.92 (28.8%)
11	F	Cauc	60	23.8	70.95 (33.6%)	26.92 (12.7%)	97.87 (46.3%)
12	F	Cauc	54	25.8	68.11 (32.2%)	*15.76 (7.5%)	83.87 (39.7%)
13	F	AA	32	29.2	53.84 (25.5%)	14.41 (6.8%)	68.25 (32.2%)
14	M	Cauc	31	27.4	90.23 (42.7%)	18.12 (8.6%)	108.35 (51.3%)
15	F	AA	42	31.3	76.66 (36.6%)	18.05 (8.5%)	94.71 (44.8%)
16	F	Asian	24	22.0	38.99 (18.5%)	16.11 (7.6%)	55.10 (26.1%)
17	F	Cauc	29	21.8	49.69 (23.5%)	36.17 (17.1%)	85.86 (40.7%)
18	F	Asian	29	20.1	75.19 (35.6%)	21.32 (10.1%)	96.51 (45.7%)
19	F	Asian	33	19.3	52.17 (24.7%)	13.33 (6.3%)	65.50 (31.0%)
20**	M	Asian	38	20.9	*38.76 (18.4%)*	*19.31 (9.1%)*	*58.07 (27.5%)*
Average	70%	45:35:20	41.6	25.5	52.7 ± 19.4	19.6 ± 6.7	72.3 ± 20.5
	F	Ca:AA:As ***			(25.4% ± 9.1%)	(9.0% ± 3.4%)	(34.3% ± 9.7%)

*incomplete 16 hour urine collection, but values included in further calculations.

**high baseline invalidates data.

***45% Cauc, 35% AA, 20% Asian

Table S2. PPI Phase demographics. BSE supplement dose ≈ 370 μmol glucoraphanin.

Participant	Sex	Race	Age (yrs)	Weight (kg)	Height (m)	BMI
1	m	Latino	31.5	78.00	1.78	24.62
2	m	White/Hispanic	26.5	65.77	1.72	22.29
3	f	Asian	29.8	52.16	1.50	23.28
4	f	African American	66.6	86.18	1.67	30.95
5	m	Hispanic	29.5	77.11	1.77	24.70
6	f	Asian	28.5	42.64	1.56	17.52
7	f	African American	48.7	69.85	1.57	28.32
8	f	Indian	22.7	58.97	1.50	26.31
9	m	Caucasian	28.7	79.38	1.82	24.07
10	f	Caucasian/Asian	25.2	62.14	1.57	25.19
11	f	Hispanic	46.0	73.94	1.60	29.06
12	m	Caucasian	28.2	73.30	1.77	23.40
13	m	Caucasian	31.8	70.31	1.74	23.16
14	m	African American	28.2	79.38	1.78	25.08
15	f	Asian	25.2	55.79	1.60	21.93
16	m	Asian	41.7	81.65	1.74	26.90

Table S3. Gene expression loadings by component after averaging triplicate determinations made at the first and second post-intervention blood draws, compared to those made at baseline. Bolded data represent gene expressions that load negatively (<0.30) or positively (>0.30) on a component. Total variability explained by three components = 70%. An anti-inflammatory gene's contributions (IL-10) group strongly in two components. The contributions of four genes (HSP27, NQ01, GCLC, s1c7a11) were unexplained by these components.

**Gene**	**Principal components (eigenvectors)**			
	**[1]Component 1**	**[2]Component 2**	**[3]Component 3**	**Unexplained**
**% variability explained**	*32%*	*26%*	*12%*	
**IL-6**	**0.4139**	*-0.0433*	*-0.0273*	*0.08294*
**IL-8**	**0.4317**	*-0.006*	*-0.0586*	*0.02140*
**IL-10**	**0.3049**	*-0.1431*	**0.3213**	*0.12980*
**Cox2**	**0.4291**	*-0.0331*	*-0.0034*	*0.03108*
**NQO1**	*-0.0795*	*0.1842*	*0.1329*	**0.62480**
**HO-1**	*0.0058*	**0.3666**	*-0.0841*	**0.45530**
**GCLC**	*-0.065*	*0.2273*	*0.2157*	**0.33720**
**GCLM**	*-0.0341*	**0.4158**	*-0.0565*	*0.23880*
**IL-1β**	**0.4279**	*0.0064*	*-0.0728*	*0.03920*
**HSP70**	*-0.03*	**0.3384**	*0.0343*	**0.39280**
**HSP27**	*0.0536*	*0.1658*	*0.1779*	**0.61820**
**IL-2**	*-0.0774*	*-0.104*	**0.4555**	*0.25350*
**SOD2**	**0.387**	*0.1905*	*0.0745*	*0.04851*
**Catalase**	*-0.0085*	*0.0299*	**0.3865**	**0.33330**
**HDAC3**	*-0.0166*	*0.0371*	**0.4417**	*0.12200*
**SLC7A11**	*-0.0027*	*0.2161*	*0.2353*	**0.35240**
**AKR1c1**	*0.0403*	**0.3313**	*-0.0046*	**0.49010**
**IFNγ**	*0.0926*	**0.3282**	*0.0017*	**0.47260**
**AKR1b10**	*-0.0156*	**0.355**	*-0.1193*	**0.50130**
**SMPD1**	*0.0042*	*-0.0455*	**0.391**	**0.42730**

[1]Component 1: IL-6, IL-8, (IL-10), Cox2, IL-1β, SOD2
[2]Component 2: H0-1, GCLM, AKR1c1, IFNγ, AKR1b10, HSP70
[3]Component 3: (IL-10), IL-2, Catalase, HDAC3, SMPD1

Figure S1. Twenty-four hour SF metabolite excretion following a single oral dose of BSE containing active myrosinase: The first 8 h. (■), and the next 16 h. (■).

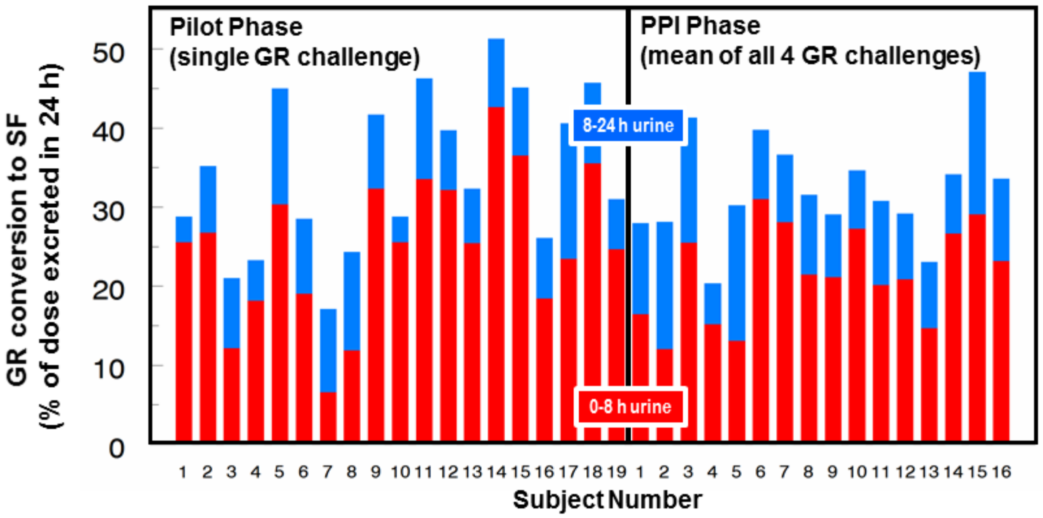

Figure S2. Relationship between self-identified ethnicity and bioavailability. Excretion of sulforaphane (SF) metabolites following a single oral dose of BSE containing glucoraphanin (GR) and active myrosinase.

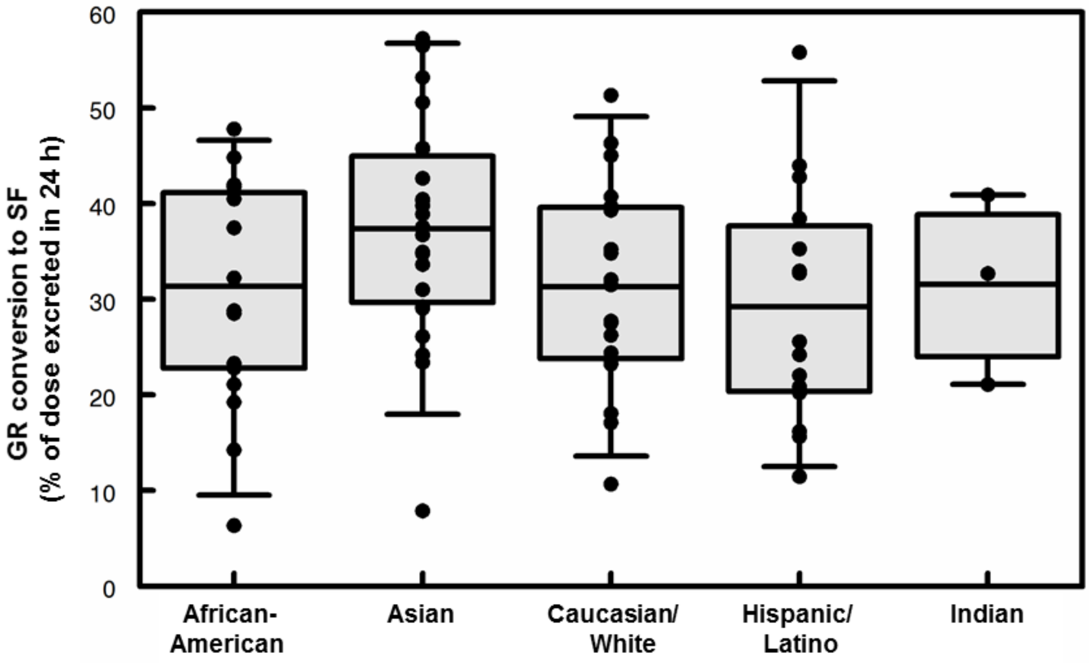

Figure S3. Relationship between age and bioavailability. Excretion of sulforaphane (SF) metabolites following a single oral dose of BSE containing glucoraphanin (GR) and active myrosinase.

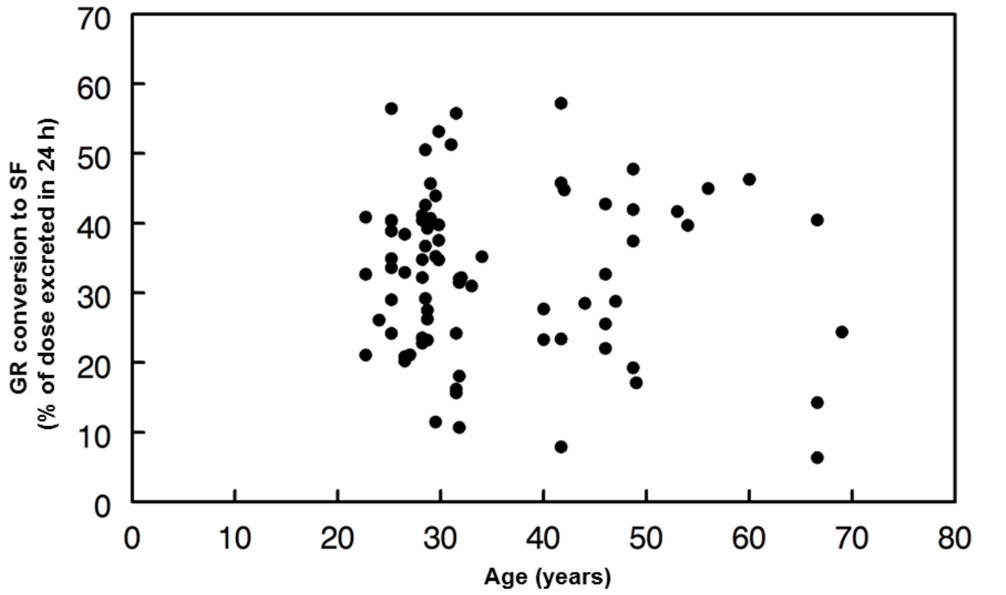

Figure S4. Relationship between body mass index (BMI) and bioavailability. Excretion of sulforaphane (SF) metabolites following a single oral dose of BSE containing glucoraphanin (GR) and active myrosinase.

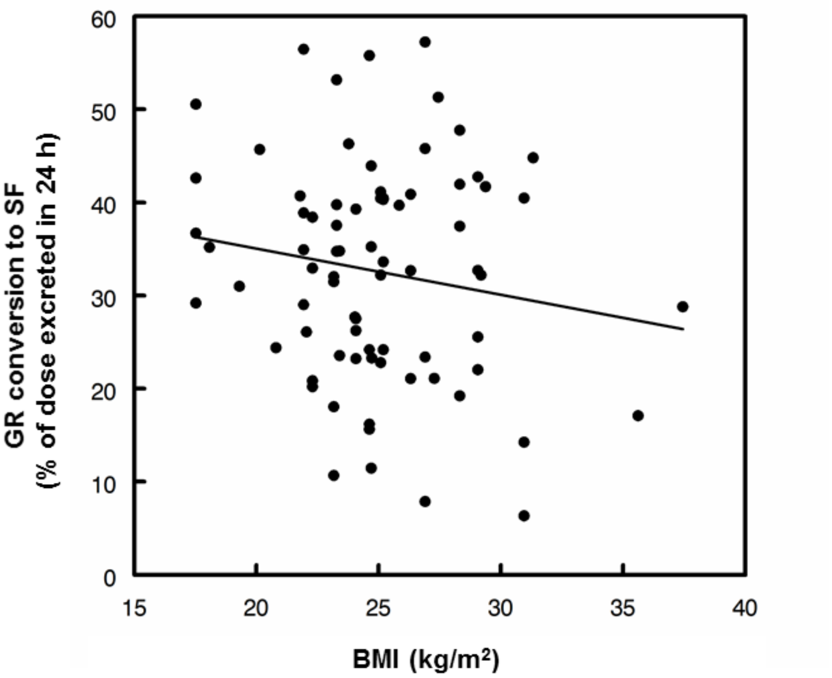

Figure S5. Effect of prior self-reported vegetable consumption on 24h. excretion of sulforaphane (SF) metabolites following a single oral dose of BSE containing glucoraphanin (GR) and active myrosinase.

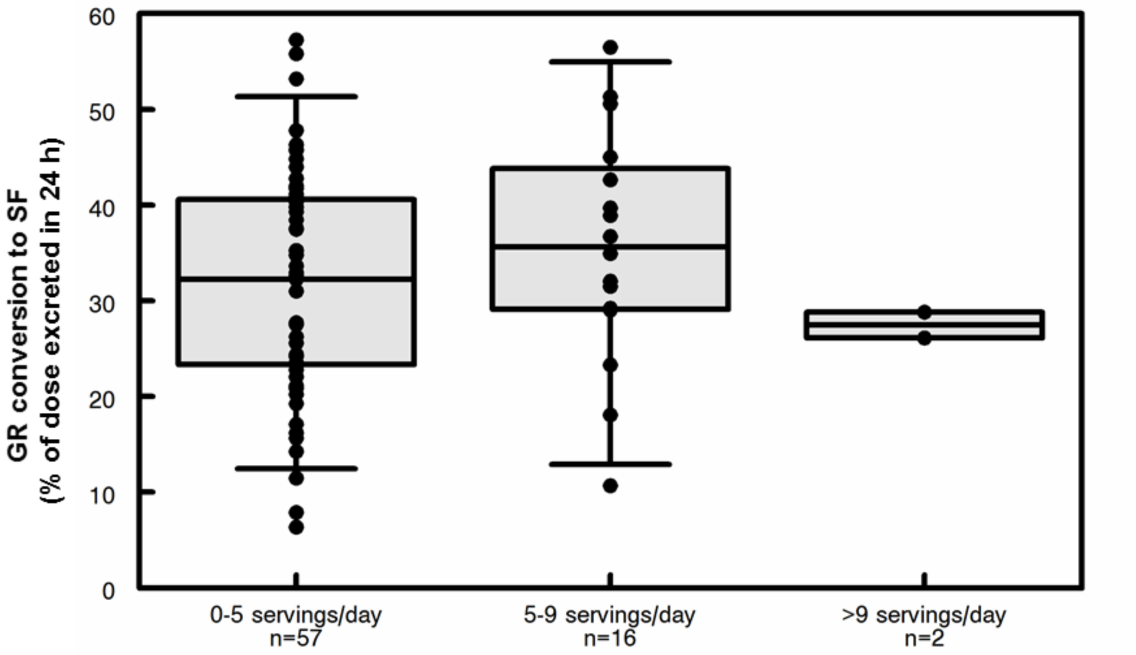

Figure S6. Relationship between self-reported (**A**) bowel movement frequency and (**B**) bowel movement quality (PPI Phase, only), on 24 h excretion of sulforaphane (SF) metabolites following a single oral dose of BSE containing glucoraphanin (GR) and active myrosinase.

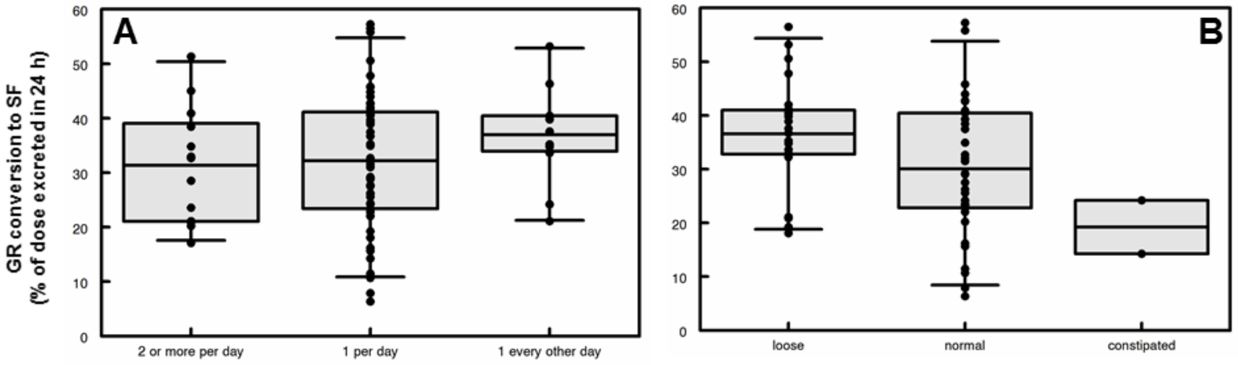

Figure S7. Mean gene amplification: 20 genes for each of 16 subjects (with their medication/supplementation status), grouped according to PCA results.

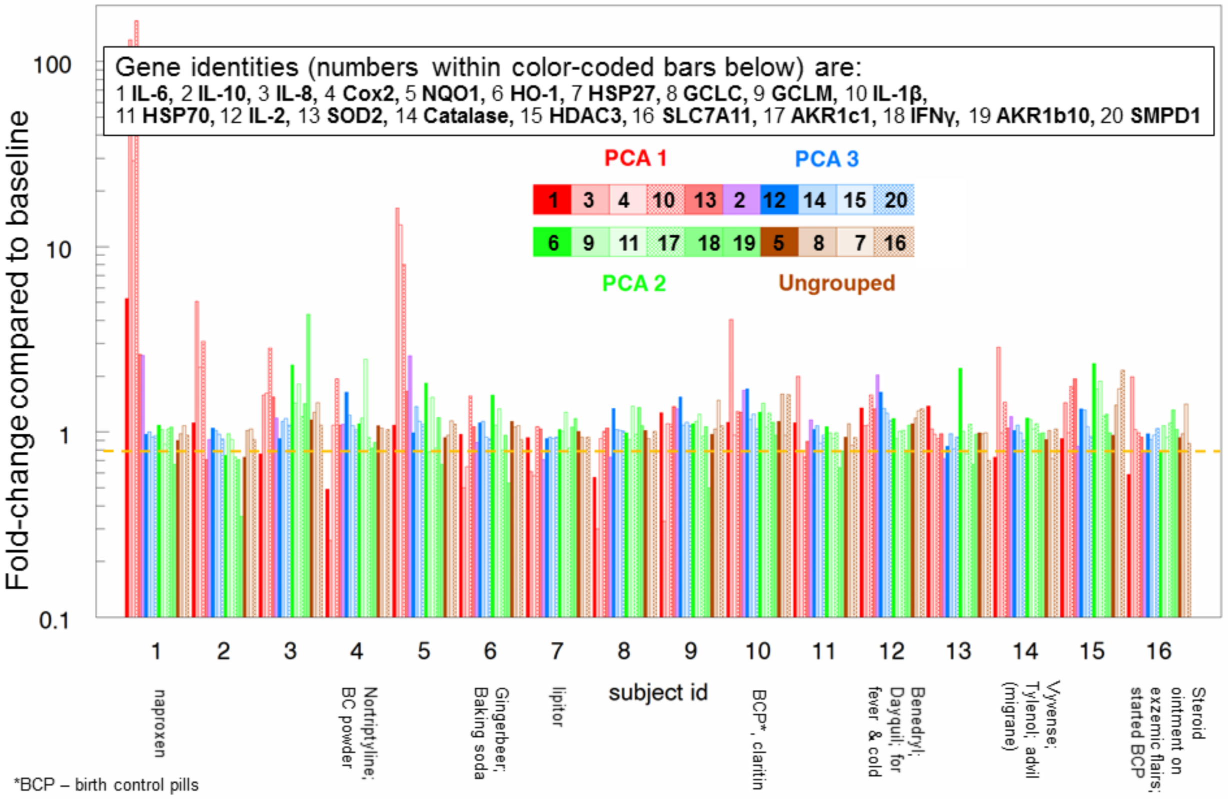

*BCP – birth control pills

Figure S8. Principle components analysis (PCA) on combined PBMC data (computed on mean following single pre- and post-omeprazole delivery of an oral BSE dose containing GR and active myrosinase). Components 1 and 2 explain 58% of variability: Component 1: IL-6, IL-8, IL-10, COX2, IL-1B, SOD2; Component 2: HO-1, GCLM, AKR1c1, IFNγ, AKR1b10; HSP70. *Note that Subject 1 had extraordinarily high IL-1β, IL-8, and COX2 gene expression at both time periods that went into the average values used to create eigenvalues. These 3 genes were all in Component 1 in the PCA analysis.

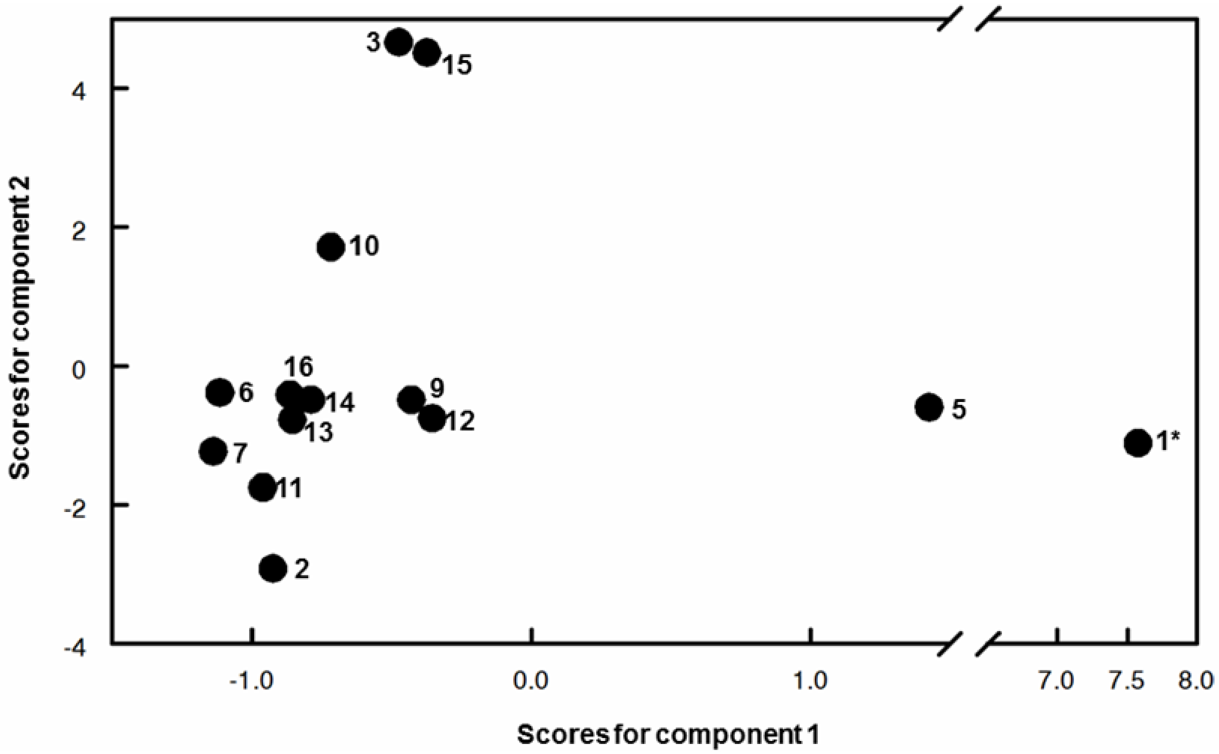

Figure S9. Gene amplification and sulforaphane bioavailability. Mean excretion of sulforaphane (SF) metabolites following single pre- to post-omeprazole delivery of an oral dose of BSE containing GR and active myrosinase. Based on individual linear regressions, '*r*' and '*p*' values for correlation between conversion and fold-amplification are provided immediately below the descriptor of each gene. **(A)** Inflammation- & immune-related; **(B)** cytoprotective, detoxifying & antioxidant; **(C)** minor or non-explanatory.

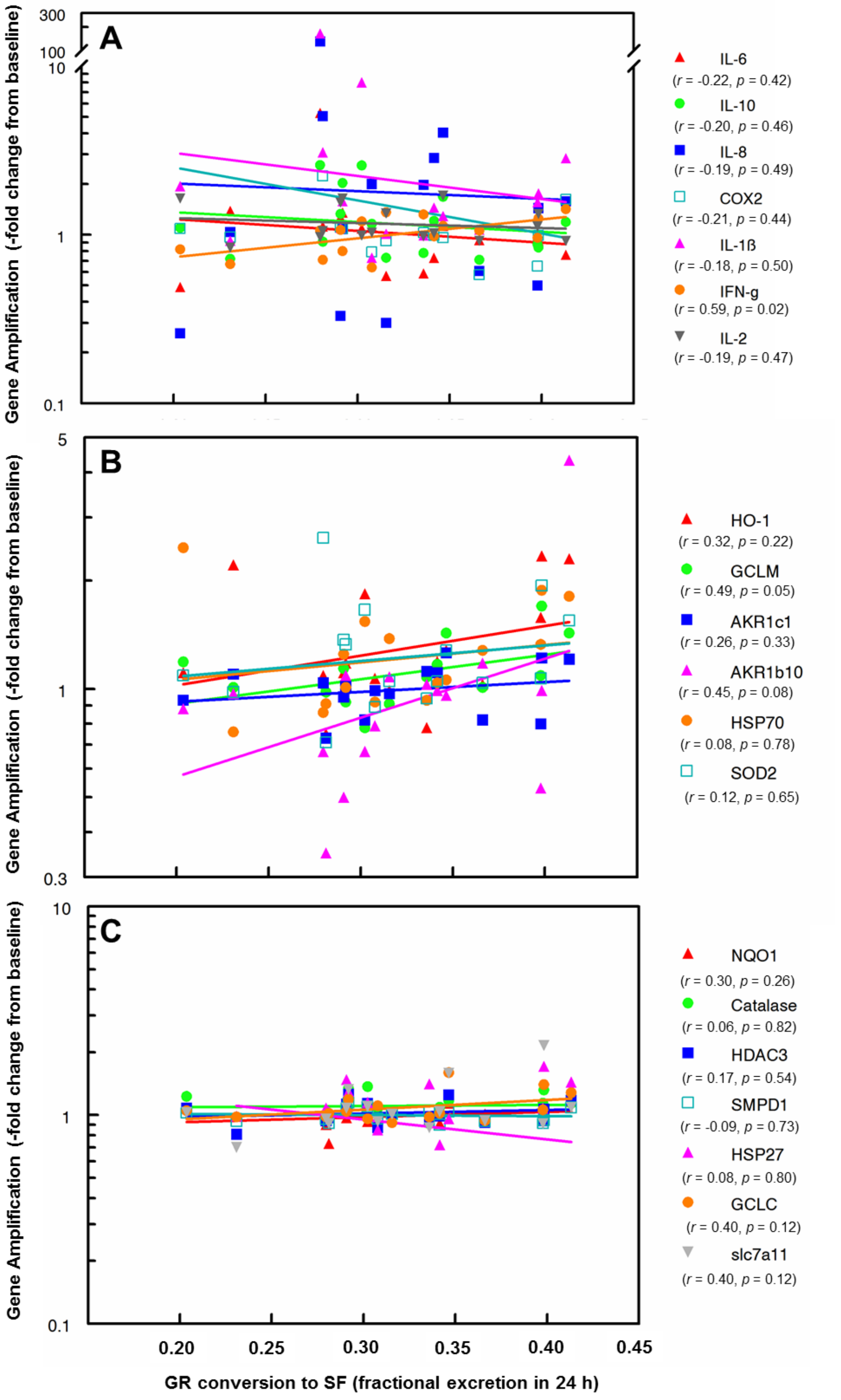

Figure S10. PCA loadings and sulforaphane bioavailability by age of subject. PC1(▲), PC2 (●), PC3(■). Loadings for a 67 and a 23 year old subject were not calculated due to missing data.

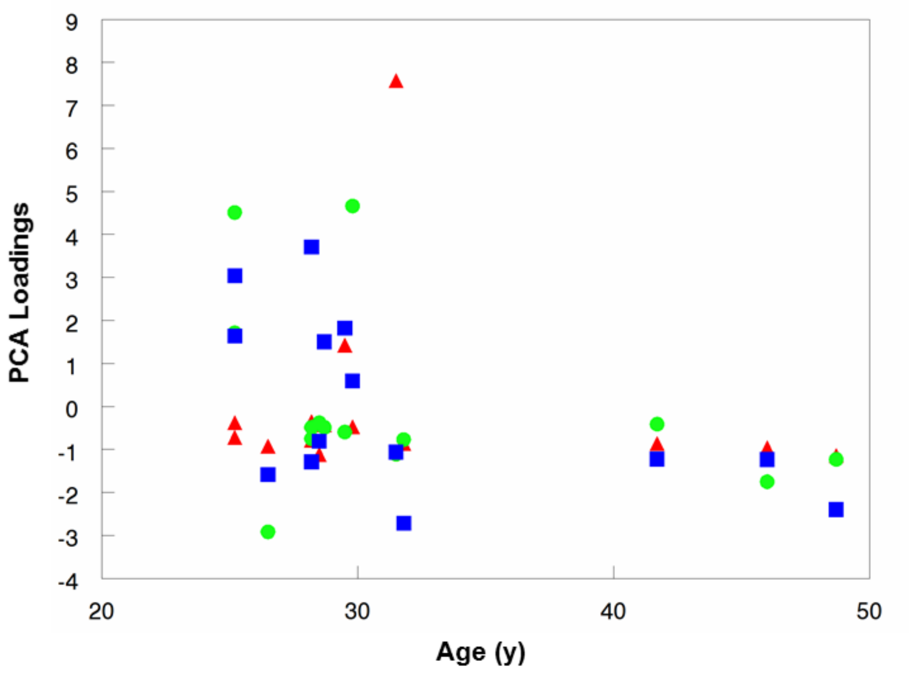

Figure S11. PCA loadings and sulforaphane bioavailability by self-identified ethnicity of subject. PC1(▲), PC2 (●), PC3(■). Loadings for one African American and one Indian subject were not calculated due to missing data.

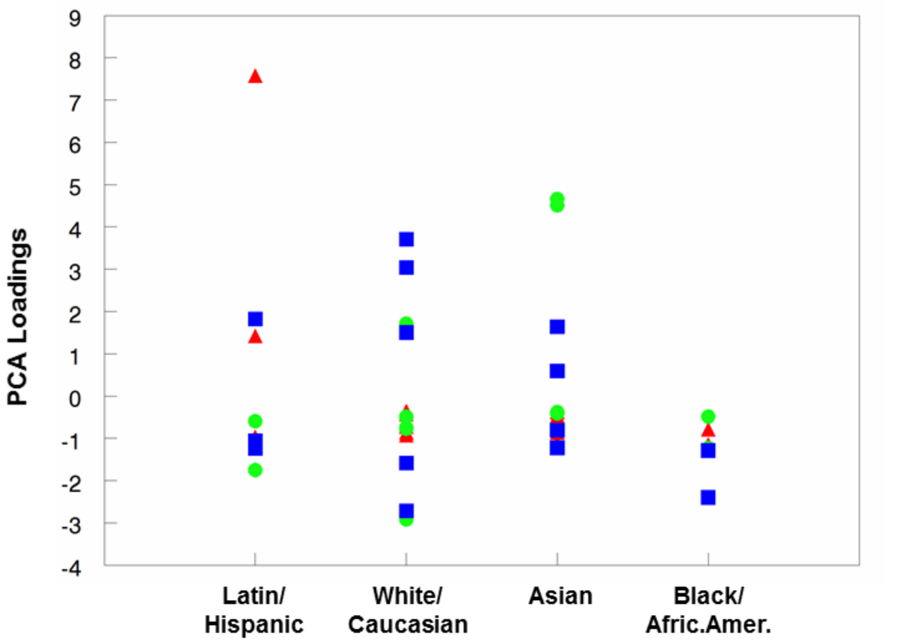

Figure S12. PCA loadings and sulforaphane bioavailability by sex of subject. PC1(▲), PC2 (●), PC3(■).

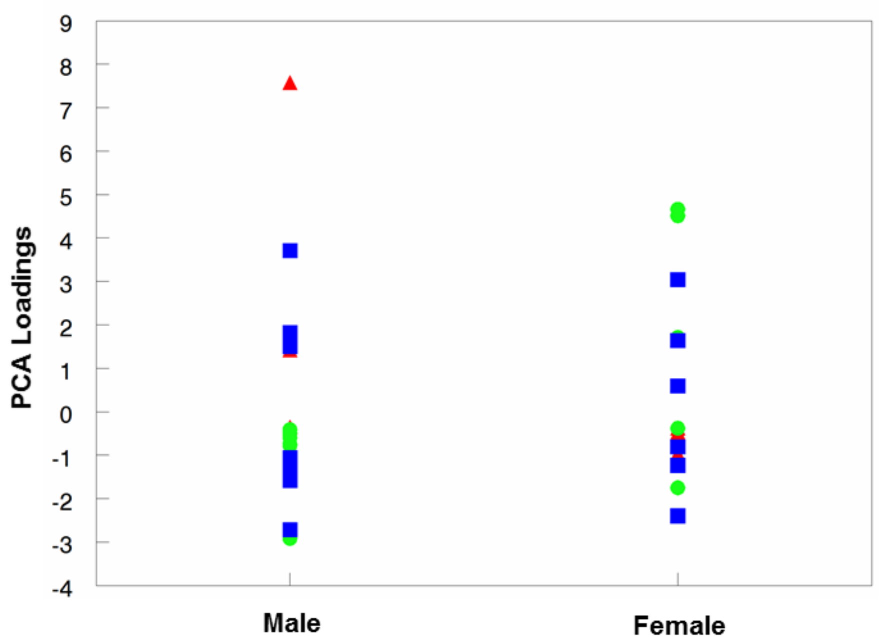

Figure S13. PCA loadings and sulforaphane bioavailability by self-identified body mass of subject. PC1(▲), PC2 (●), PC3(■). Loadings for an 86 and a 59 kg subject were not calculated due to missing data.

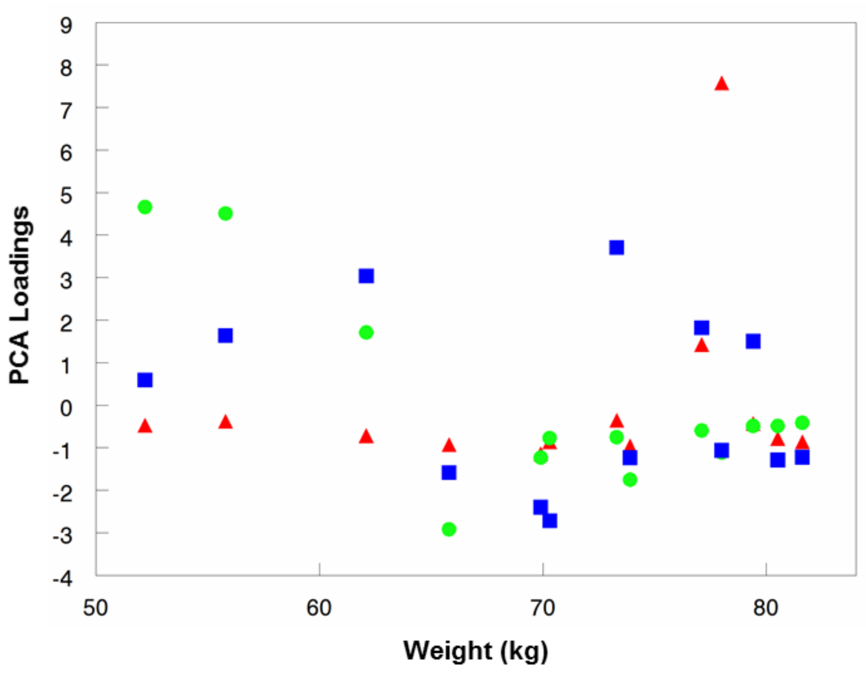

Supplement: Supplementary file 1 [file nutrients-11-01489-s001.pdf]
